# Chlorido­(2,2′-{[2-(1-methyl-1*H*-imidazol-2-yl-κ*N* ^3^)imidazolidine-1,3-diyl-κ*N*]bis­(methyl­ene)}bis­(1-methyl-1*H*-imidazole-κ*N* ^3^))copper(II) perchlorate

**DOI:** 10.1107/S2056989019004055

**Published:** 2019-04-02

**Authors:** Diego da Silva Padilha, Marciela Scarpellini

**Affiliations:** a Instituto de Química, Universidade Federal do Rio de Janeiro, Av. Athos da, Silveira Ramos, 149, Bl. A, Lab. 628a. CEP 21941-909, Rio de Janeiro, RJ, Brazil

**Keywords:** crystal structure, copper complex, imidazolidine ligand

## Abstract

In the crystal structure of the title complex, [CuCl(C_17_H_24_N_8_)]ClO_4_, the copper(II) metal exhibits an N_4_Cl penta­coordinate environment in a distorted square-pyramidal geometry. Coordination to the metal centre occurs through the three 1-methyl­imidazole N atoms from the pendant groups, one amine N atom from the imidazolidine moiety and one chlorido anion. Inter­molecular inter­actions take place at two of the 1-methyl-­imidazole rings in the form of parallel-displaced π–π stacking inter­actions forming chains parallel to the *a* axis. Three O atoms of the perchlorate anion are rotationally disordered between two orientations with occupancies of 0.5.

## Abstract 2

The mol­ecular and crystal structure of a novel copper(II) complex bearing a new tetra­dentate 1-methyl-imidazole-containing imidazolidine ligand is described.

## Chemical context   

Copper ions play a key role in many natural processes, as they are found in the active site of enzymes involved in electron and O_2_ transfers, oxidation and reduction, being a target for the obtaining of biomimetic or bioinspired compounds (Stephanos & Addison, 2014 ▸). As a result of the redox characteristics of the copper ion, the versatility of ligands to which it coordinates, and the geometries it is capable of forming, copper complexes have attracted attention as catalysts for different transformations, mainly involving the activation and reduction of oxygen (Elwell *et al.*, 2017 ▸). For the hydrogen evolution reaction (HER), the obtaining of homogeneous copper catalysts is limited by the dissociation of copper(II) because of the more negative potentials required for the reduction of protons (Zhang *et al.*, 2014 ▸; Du *et al.*, 2016 ▸). However, different copper complexes have been obtained and evaluated as catalysts for HER, showing promising results (Zhang *et al.*, 2016 ▸; Haddad *et al.*, 2017 ▸; Khusnutdinova *et al.*, 2018 ▸). The use of bioinspired tripodal tetra­dentate ligands in the construction of metal complexes catalysts can provide a unique feature, the presence of *cis*-labile sites for substrate coordination that may be a requisite for its catalytic activity, facilitating electron/atom transfer processes.
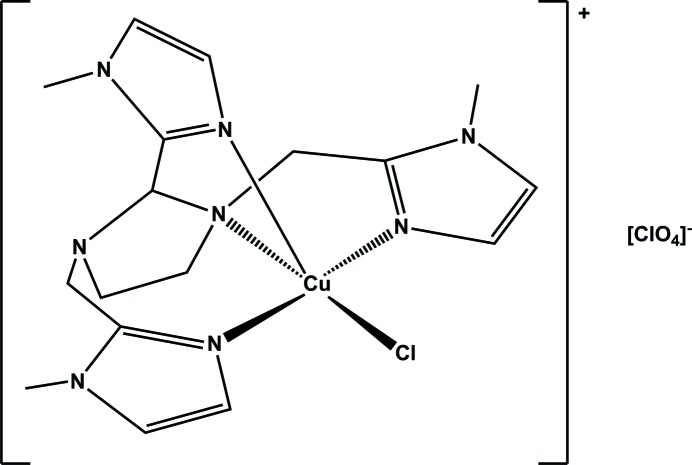


Herein, we report the mol­ecular and crystal structure of a novel mononuclear copper(II) complex bearing an imidazolidine tetra­dentate ligand, namely chlorido­(2,2′-{[2-(1-methyl-1*H*-imidazol-2-yl-κ*N* ^3^)imidazolidine-1,3-diyl-κ*N*]bis­(methylene)}bis­(1-methyl-1*H*-imidazole-κ*N* ^3^)copper(II) perchlorate, [Cu(*L*)Cl]ClO_4_. Similar complexes obtained with penta­dentate ligands derived from the imidazolidine ring opening were previously reported (Cisnetti *et al.*, 2007 ▸; Garcia *et al.*, 2015 ▸), but to the best of our knowledge, this is the first example of a copper complex bearing an imidazolidine ligand with three 1-methyl-imidazole side arms.

## Structural commentary   

**Figure 1:**
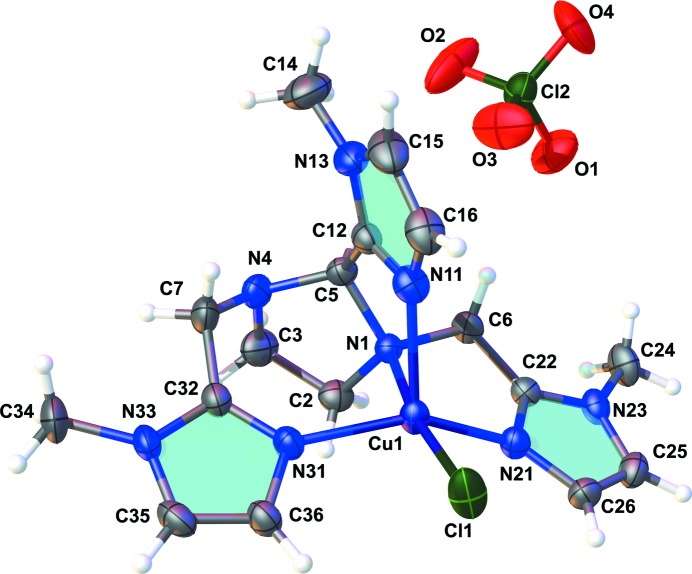
The structure of the title compound with displacement ellipsoids drawn at the 50% probability level. Only one component of the disordered oxygen atoms of the perchlorate anion is shown.

The title complex crystallizes in the monoclinic system, space group *P*21/*n*. The asymmetric unit comprises one complex cation and one disordered perchlorate anion (Fig. 1 ▸). The copper(II) ion has an N_4_Cl penta­coordinated environment formed by one ligand mol­ecule and one chlorido ion. Coord­ination of the ligand to the metal centre occurs through the three 1-methyl-imidazole nitro­gen atoms (N_Me-im_) and one of the tertiary amine nitro­gen atoms from the imidazolidine moiety (N_am_). A distorted square-pyramidal geometry is observed (τ = 0.39), with the basal plane composed of the chlorido ion, the amine nitro­gen and the two equivalent 1-methyl-imidazole nitro­gen atoms N11/N21. The third 1-methyl-­imidazole nitro­gen N31 occupies the apical position. Distortion of the geometry is evidenced by the bond angles in the coordin­ation sphere, ranging from 77.43 (11) to 113.64 (12)° and 147.65 (12) to 171.21 (8)° for the *cis* and *trans* angles, respectively. This highly distorted square-pyramidal geometry may arise from the formation of a seven-membered chelate ring (Cu1/N1/C5/N4/C7/C32/N31) that is less tensioned than the four or five-membered rings, allowing a more flexible arrangement. As a consequence of the geometry distortion, the copper(II) ion lies 0.2565 (13) Å above the Cl1/N21/N1/N11 basal plane towards the apical position. Square-pyramidal copper complexes exhibiting a smaller geometry distortion tends to show a slighter displacement of the metal centre from the basal plane. In the similar [Cu(bpqa)Cl]^+^ (τ = 0.16) and [Cu(tmqa)Cl]^+^ (τ = 0.06) complexes [bpqa = 1-(pyridin-2-yl)-*N*-(pyridin-2-ylmeth­yl)-*N*-(quinolin-2-ylmeth­yl)methan­amine; tmqa = tris­(quinolin-2-ylmeth­yl)amine; Wei *et al.*, 1994 ▸], the copper(II) ion is 0.189 (2) and 0.045 (3) Å above the basal plane, respectively. For the complexes [Cu(*L*)(ONO)]^+^ (τ = 0.27; *L* = [bis­(2-methyl­imidazol-2-yl)meth­yl][2-(pyridyl-2-yl)eth­yl]amine; Scarpellini *et al.*, 2004*a* ▸) and [Cu(Hhis-im_2_)Cl]^+^ {τ = 0.31; Hhis-im_2_ = 2-(1*H*-imidazol-4-yl)-*N*,*N*-bis­[(1-methyl-1*H*-imidazol-2-yl)meth­yl]ethanamine; Higa *et al.*, 2007 ▸}, the copper-to-plane distances are 0.1761 (1) and 0.23918 (10) Å, respectively.

The Cu1—Cl1 bond length is 2.2698 (10) Å, being the longest in the coordination sphere. This value is in good agreement with the Cu—Cl bond lengths of 2.2742 (11) and 2.2690 (12) Å reported for the complexes [Cu(pmea)Cl]^+^ [pmea = 2-(pyridin-2-yl)-*N*,*N*-bis­(pyridin-2-ylmeth­yl)ethan­amine] and [Cu(pmap)Cl]^+^ [pmap = 2-(pyridin-2-yl)-*N*-(2-(pyridin-2-yl)eth­yl)-*N*-(pyridin-2-ylmeth­yl)ethanamine; Schatz *et al.*, 2001 ▸]. For the complexes [Cu(hismimi)Cl_2_] and [Cu(hismima)Cl_2_] {hismimi = 2-(1*H*-imidazol-4-yl)-*N*-[(1-methyl-1*H*-imidazol-2-yl)methyl­ene]ethanamine; hismima = 2-(1*H*-imidazol-4-yl)-*N*-[(1-methyl-1*H*-imidazol-2-yl)meth­yl]ethanamine; Scarpellini *et al.*, 2003 ▸}, the Cu—Cl bond lengths range from 2.2882 (11) to 2.2930 (11) Å for the Cl atoms in the basal plane and from 2.5705 (10) to 2.5789 (10) Å for the Cl atoms occupying the apical position.

The Cu—N_Me-im_ bond lengths in the title compound range from 1.976 (3) to 2.173 (3) Å, the longest one being formed by the 1-methyl-­imidazole nitro­gen N11. For the Cu—N_am_ bond, a distance of 2.137 (3) Å was found. Similar values were reported for the 1-methyl-imidazole-containing complexes [Cu(Hhis-im_2_)Cl]^+^ (Higa *et al.*, 2007 ▸), [Cu(hismima)(his)]^+^ {hismima = 2-(1*H*-imidazol-4-yl)-*N*-[(1-methyl-1*H*-imidazol-2-yl)meth­yl]ethanamine; his = histamine; Scarpellini *et al.*, 2001 ▸}, [Cu_2_(hismima)_2_Cl_2_]_2_^2+^ (Scarpellini *et al.*, 2004*b* ▸), [Cu(pymimi)Cl_2_] and [Cu(pymima)Cl_2_] {pymimi = [2-(pyridyl-2-yl)eth­yl][(1-methyl­imidazol-2-yl)meth­yl]imine; pymima = [2-(pyridyl-2-yl)eth­yl][(1-methyl­imidazol-2-yl)meth­yl]amine; Ferre *et al.*, 2017 ▸}.

## Supra­molecular features   

**Figure 2:**
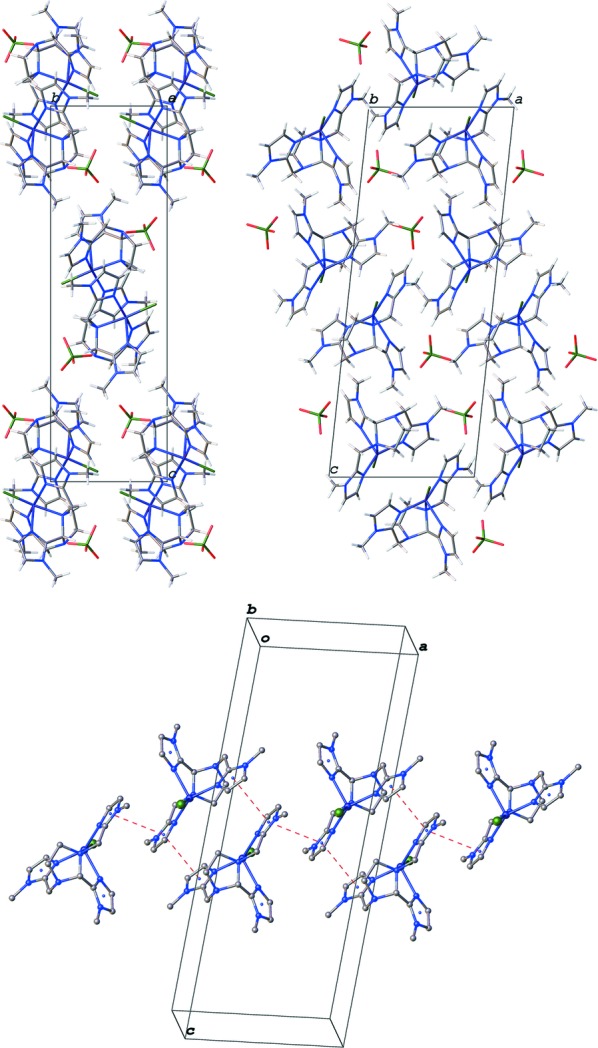
Crystal packing (viewed perpendicular to (100), top left, and (010), top right) and inter­molecular π–π stacking inter­actions (dashed lines, bottom) in the structure of the title compound.

Inter­molecular contacts in the title compound occur through π–π stacking inter­actions (Fig. 2 ▸) involving two 1-methyl-imidazole rings (N21/C22/N23/C25/C26 and N31/C32/N33/C35/C36), forming chains that propagate parallel to the *a* axis. The inter­centroid distances are 3.690 (2) and 3.761 (2) Å, the centroid-to-plane distances are 3.4719 (15) and 3.6240 (15) Å, and the parallel shifts are 1.250 (6) and 1.008 (7) Å.

## Features of related complexes   

In penta­coordinated copper(II) complexes containing tripodal N_4_ donor ligands similar to the title compound, the Cu—Cl bond length seems to be directly related to the type and degree of geometry distortion around the metal centre. In complexes exhibiting a square-pyramidal geometry, as in the title compound, the Cu—Cl bond length has a range of 2.27–2.29 Å. For complexes in a trigonal–bipyramidal geometry, the Cu—Cl distance is around 2.23 Å (Karlin *et al.*, 1982 ▸; Oberhausen *et al.*, 1990 ▸; Wang *et al.*, 1995 ▸). This difference may be related to the ligand spatial orientation, resulting from the geometric arrangements around the metal centre. The trigonal–bipyramidal geometry imposes a vertical positioning of the coordinated ligand rings parallel to the axial direction, which minimizes the repulsion between the electronic clouds of the chloride ion and the tripodal ligand. This arrangement allows a greater approach of the chloride ion to the metal centre and consequently a shorter bond distance. In the case of complexes in a square-pyramidal geometry, the coordinated rings are oriented parallel to the basal plane, increasing the chloride/ligand repulsion effect, which makes the Cu—Cl bond more elongated. Curiously, copper complexes in both geometries with tripodal ligands showing steric hindrance exhibit inter­mediate Cu—Cl bond distances among those found for complexes with non-hindered ligands on square-pyramidal and trigonal–bipyramidal geometries, indicating a balance of repulsive and stabilizing chloride/ligand inter­actions that is geometry independent (Wei *et al.*, 1994 ▸; Jitsukawa *et al.*, 2001 ▸).

## Synthesis and crystallization   

**2,2′-{[2-(1-Methyl-1** ***H*** **-imidazol-2-yl)imidazolidine-1,3-di­yl]bis­(methyl­ene)}bis­(1-methyl-1** ***H*** **-imidazole)**, ***L***: The new ligand **L** was synthesized by condensation reaction between *N* ^1^,*N* ^2^-bis­[(1-methyl-1*H*-imidazol-2-yl)meth­yl]ethane-1,2-di­amine (Neves *et al.*, 1997 ▸) (1.8939 g, 7.63 mmol) and 1-methyl-2-imidazole­carboxaldehyde (0.8401 g, 7.63 mmol) in ethano­lic media (40 ml). The reaction mixture was stirred for 24 h at room temperature, when the solvent was removed by rota-evaporation. To the resulting white solid, 40 ml of ethyl ether were added, and the mixture was stirred at room temperature for 24 h. After removal of the solvent under reduced pressure, the resulting white solid was recrystallized from acetone. Yield after recrystallization: 2.4 g (92%). ^1^H NMR (500 MHz, DMSO) δ 7.16 (*d*, *J* = 0.8 Hz, 1H), 7.01 (*d*, *J* = 1.1 Hz, 2H), 6.84 (*d*, *J* = 1.1 Hz, 1H), 6.73 (*d*, *J* = 1.2 Hz, 2H), 4.20 (*s*, 1H), 3.74 (*s*, 3H), 3.63 (*d*, *J* = 13.5 Hz, 2H), 3.43 (*d*, *J* = 13.5 Hz, 2H), 3.33 (s, 6H), 2.96–2.87 (*m*, 2H), 2.75–2.67 (*m*, 2H) p.p.m. ^13^C NMR (126 MHz, DMSO) δ 144.89, 144.45, 127.16, 126.75, 124.13, 122.18, 82.92, 50.45, 33.37, 32.19 p.p.m..

**[Cu(** ***L*** **)Cl]ClO_4_**: The synthesis was achieved by reacting CuCl_2_·2H_2_O (0.1708 g, 1 mmol) and the ligand **L** (0.3403 g, 1 mmol) in ethano­lic media, at room temperature. Recrystallization of the obtained amorphous green solid in aceto­nitrile solution at room temperature yielded 0.152 g (28%) of green single crystals after one day. IR (cm^−1^, KBr): 3460 (ν O—H), 3160–3130 (ν C—H_arom_), 2972–2854 (ν C—H_ali_), 1636–1419 (ν C=N/C=C_ring_), 1285 (ν *C*—N_amine_), 1096/623 (ν Cl—O), 771 (δ C—H_arom_), 502 (ν Cu—N_amine_), 291 (ν Cu—Cl).

## Refinement   

Crystal data, data collection and structure refinement details are summarized in Table 1 ▸. The perchlorate anion is rotationally disordered over two orientations sharing the O1 oxygen atom with site occupancy factors of 0.5. The two disordered positions were refined by applying SADI restraints on the Cl–O bond lengths and O⋯O separations. The *U*_ij_ parameters of the Cl2 atom were restrained to an approximate isotropic behaviour.

## Supplementary Material

Crystal structure: contains datablock(s) I. DOI: 10.1107/S2056989019004055/rz4029sup1.cif

Structure factors: contains datablock(s) I. DOI: 10.1107/S2056989019004055/rz4029Isup2.hkl

CCDC reference: 1905520

Additional supporting information:  crystallographic information; 3D view; checkCIF report

## Figures and Tables

**Table 1:** Experimental details

Crystal data	
Chemical formula	[CuCl(C_17_H_24_N_8_)]ClO_4_
*M*_r_	538.88
Crystal system, space group	Monoclinic, *P*2_1_/*n*
Temperature (K)	288
*a*, *b*, *c* (Å)	10.2904 (4), 8.1336 (3), 26.317 (1)
β (°)	96.170 (1)
*V* (Å^3^)	2189.92 (14)
*Z*	4
Radiation type	Mo *K*α
μ (mm^−1^)	1.29
Crystal size (mm)	0.25 × 0.16 × 0.09

Data collection	
Diffractometer	Bruker D8 Venture
Absorption correction	Multi-scan (*SADABS*; Bruker, 2015 ▸)
*T*_min_, *T*_max_	0.656, 0.745
No. of measured, independent and observed [*I* > 2σ(*I*)] reflections	41042, 4471, 3704
*R*_int_	0.069
(sin θ/λ)_max_ (Å^−1^)	0.625

Refinement	
*R*[*F* ^2^ > 2σ(*F* ^2^)], *wR*(*F* ^2^), *S*	0.050, 0.102, 1.12
No. of reflections	4471
No. of parameters	319
No. of restraints	63
H-atom treatment	H-atom parameters constrained
Δρ_max_, Δρ_min_ (e Å^−3^)	0.52, −0.53

Computer programs: *APEX2* (Bruker, 2015 ▸), *SAINT* (Bruker, 2015 ▸, *SHELXT* (Sheldrick, 2015*a* ▸), *SHELXL* (Sheldrick, 2015*b* ▸) and *OLEX2* (Dolomanov *et al.*, 2009 ▸).

## supplementary crystallographic information

### Crystal data

**Table:**

[CuCl(C_17_H_24_N_8_)]ClO_4_	*F*(000) = 1108
*M**_r_* = 538.88	*D*_x_ = 1.634 Mg m^−^^3^
Monoclinic, *P*2_1_/*n*	Mo *K*α radiation, λ = 0.71073 Å
*a* = 10.2904 (4) Å	Cell parameters from 117 reflections
*b* = 8.1336 (3) Å	θ = 2.9–22.7°
*c* = 26.317 (1) Å	µ = 1.29 mm^−^^1^
β = 96.170 (1)°	*T* = 288 K
*V* = 2189.92 (14) Å^3^	Block, clear light green
*Z* = 4	0.25 × 0.16 × 0.09 mm

### Data collection

**Table:**

Bruker D8 Venture diffractometer	3704 reflections with *I* > 2σ(*I*)
φ and ω scans	*R*_int_ = 0.069
Absorption correction: multi-scan (SADABS; Bruker, 2015)	θ_max_ = 26.4°, θ_min_ = 2.2°
*T*_min_ = 0.656, *T*_max_ = 0.745	*h* = −12→12
41042 measured reflections	*k* = −10→10
4471 independent reflections	*l* = −32→32

### Refinement

**Table:**

Refinement on *F*^2^	Primary atom site location: dual
Least-squares matrix: full	Hydrogen site location: inferred from neighbouring sites
*R*[*F*^2^ > 2σ(*F*^2^)] = 0.050	H-atom parameters constrained
*wR*(*F*^2^) = 0.102	*w* = 1/[σ^2^(*F*_o_^2^) + (0.0274*P*)^2^ + 4.7286*P*] where *P* = (*F*_o_^2^ + 2*F*_c_^2^)/3
*S* = 1.12	(Δ/σ)_max_ = 0.001
4471 reflections	Δρ_max_ = 0.52 e Å^−^^3^
319 parameters	Δρ_min_ = −0.53 e Å^−^^3^
63 restraints	

### Special details

**Table:**

Geometry. All esds (except the esd in the dihedral angle between two l.s. planes) are estimated using the full covariance matrix. The cell esds are taken into account individually in the estimation of esds in distances, angles and torsion angles; correlations between esds in cell parameters are only used when they are defined by crystal symmetry. An approximate (isotropic) treatment of cell esds is used for estimating esds involving l.s. planes.
Refinement. All H atoms were placed geometrically and refined using a riding atom approximation, with C–H = 0.93–0.98 Å, and with *U*_iso_(H) = 1.2*U*_eq_(C) or 1.5*U*_eq_(C) for methyl H atoms. A rotating model was used for the methyl groups.

### Fractional atomic coordinates and isotropic or equivalent isotropic displacement parameters (Å^2^)

**Table:**

	*x*	*y*	*z*	*U*_iso_*/*U*_eq_	Occ. (<1)
Cu1	0.18398 (4)	0.35492 (5)	0.56226 (2)	0.02658 (12)	
Cl2	0.61349 (10)	0.81015 (12)	0.66768 (4)	0.0394 (2)	
Cl1	0.20109 (10)	0.10125 (11)	0.52765 (4)	0.0442 (3)	
N21	0.2633 (3)	0.4598 (3)	0.50530 (11)	0.0276 (6)	
N1	0.1935 (3)	0.6031 (3)	0.58904 (10)	0.0237 (6)	
N31	0.0181 (3)	0.3032 (4)	0.59528 (11)	0.0298 (6)	
N23	0.3807 (3)	0.6599 (4)	0.47829 (11)	0.0293 (6)	
N11	0.3159 (3)	0.3367 (4)	0.63224 (12)	0.0324 (7)	
N33	−0.1361 (3)	0.2601 (4)	0.64552 (11)	0.0332 (7)	
N13	0.3635 (3)	0.4347 (4)	0.70991 (11)	0.0362 (7)	
N4	0.0918 (3)	0.6184 (4)	0.66510 (11)	0.0331 (7)	
C12	0.2988 (3)	0.4602 (4)	0.66327 (13)	0.0283 (7)	
C26	0.2978 (3)	0.4181 (5)	0.45779 (13)	0.0319 (8)	
H26	0.275354	0.321232	0.440245	0.038*	
C22	0.3146 (3)	0.6059 (4)	0.51634 (12)	0.0241 (7)	
C5	0.2183 (3)	0.6065 (4)	0.64627 (12)	0.0267 (7)	
H5	0.268323	0.705847	0.656460	0.032*	
C32	−0.0283 (3)	0.3493 (5)	0.63838 (13)	0.0307 (8)	
C6	0.3044 (3)	0.6840 (4)	0.56629 (13)	0.0286 (8)	
H6A	0.287929	0.800888	0.561970	0.034*	
H6B	0.385168	0.669535	0.588489	0.034*	
C36	−0.0650 (3)	0.1818 (4)	0.57440 (15)	0.0336 (8)	
H36	−0.057209	0.127441	0.543794	0.040*	
C25	0.3696 (4)	0.5416 (5)	0.44102 (14)	0.0341 (8)	
H25	0.405045	0.545645	0.409984	0.041*	
C35	−0.1586 (3)	0.1546 (5)	0.60512 (15)	0.0388 (9)	
H35	−0.226056	0.078382	0.599912	0.047*	
C7	0.0270 (4)	0.4682 (5)	0.67877 (14)	0.0388 (9)	
H7A	−0.044043	0.500341	0.698061	0.047*	
H7B	0.089098	0.407663	0.702043	0.047*	
C2	0.0650 (3)	0.6893 (4)	0.57597 (14)	0.0328 (8)	
H2A	0.003612	0.619174	0.555523	0.039*	
H2B	0.076571	0.790207	0.557356	0.039*	
C24	0.4503 (4)	0.8151 (5)	0.47750 (16)	0.0414 (10)	
H24A	0.390577	0.904225	0.480668	0.062*	
H24B	0.488065	0.825186	0.445837	0.062*	
H24C	0.518329	0.818347	0.505466	0.062*	
C16	0.3943 (4)	0.2272 (5)	0.66066 (16)	0.0407 (9)	
H16	0.422015	0.126906	0.648747	0.049*	
C3	0.0176 (4)	0.7253 (5)	0.62797 (15)	0.0437 (10)	
H3A	0.032731	0.839800	0.637102	0.052*	
H3B	−0.075173	0.702826	0.626991	0.052*	
C15	0.4254 (4)	0.2850 (5)	0.70806 (16)	0.0458 (10)	
H15	0.478094	0.234306	0.734412	0.055*	
C34	−0.2202 (4)	0.2733 (6)	0.68726 (17)	0.0541 (12)	
H34A	−0.169792	0.249093	0.719228	0.081*	
H34B	−0.291094	0.196567	0.681554	0.081*	
H34C	−0.254383	0.382966	0.688193	0.081*	
C14	0.3707 (5)	0.5456 (6)	0.75403 (16)	0.0577 (12)	
H14A	0.380595	0.656728	0.742796	0.087*	
H14B	0.444214	0.516161	0.777864	0.087*	
H14C	0.291865	0.536478	0.770330	0.087*	
O1	0.5896 (4)	0.8829 (4)	0.61817 (11)	0.0659 (10)	
O2	0.551 (2)	0.904 (3)	0.7022 (7)	0.089 (6)	0.5
O3	0.5625 (15)	0.6503 (12)	0.6636 (6)	0.074 (4)	0.5
O4	0.7476 (8)	0.803 (3)	0.6829 (7)	0.093 (6)	0.5
O4'	0.7405 (11)	0.851 (3)	0.6894 (7)	0.109 (7)	0.5
O3'	0.604 (2)	0.6372 (13)	0.6674 (8)	0.124 (8)	0.5
O2'	0.526 (2)	0.870 (3)	0.7006 (9)	0.094 (7)	0.5

### Atomic displacement parameters (Å^2^)

**Table:**

	*U*^11^	*U*^22^	*U*^33^	*U*^12^	*U*^13^	*U*^23^
Cu1	0.0292 (2)	0.0192 (2)	0.0325 (2)	−0.00422 (17)	0.00912 (17)	−0.00224 (18)
Cl2	0.0445 (5)	0.0387 (5)	0.0368 (5)	−0.0036 (4)	0.0122 (4)	−0.0021 (4)
Cl1	0.0441 (5)	0.0221 (4)	0.0698 (7)	−0.0053 (4)	0.0219 (5)	−0.0113 (4)
N21	0.0282 (15)	0.0246 (15)	0.0307 (16)	−0.0030 (12)	0.0062 (12)	−0.0021 (12)
N1	0.0239 (14)	0.0234 (14)	0.0240 (14)	0.0002 (11)	0.0038 (11)	0.0010 (11)
N31	0.0260 (15)	0.0310 (16)	0.0329 (16)	−0.0058 (12)	0.0058 (12)	−0.0013 (13)
N23	0.0258 (14)	0.0281 (15)	0.0350 (16)	0.0026 (12)	0.0077 (12)	0.0080 (13)
N11	0.0320 (16)	0.0256 (15)	0.0397 (17)	0.0030 (13)	0.0041 (13)	0.0037 (14)
N33	0.0213 (15)	0.0458 (19)	0.0336 (17)	−0.0043 (13)	0.0073 (12)	0.0035 (15)
N13	0.0349 (17)	0.0458 (19)	0.0278 (16)	−0.0012 (14)	0.0028 (13)	0.0076 (14)
N4	0.0322 (16)	0.0355 (17)	0.0328 (16)	0.0020 (13)	0.0090 (13)	−0.0078 (14)
C12	0.0237 (17)	0.0317 (19)	0.0296 (19)	−0.0019 (14)	0.0034 (14)	0.0039 (15)
C26	0.0322 (19)	0.0334 (19)	0.0302 (19)	−0.0010 (15)	0.0036 (15)	−0.0064 (16)
C22	0.0238 (16)	0.0204 (16)	0.0279 (17)	−0.0002 (13)	0.0024 (13)	0.0020 (13)
C5	0.0280 (17)	0.0263 (18)	0.0256 (17)	−0.0013 (14)	0.0016 (14)	−0.0033 (14)
C32	0.0244 (17)	0.038 (2)	0.0307 (18)	−0.0017 (15)	0.0057 (14)	0.0013 (16)
C6	0.0309 (18)	0.0213 (17)	0.0339 (19)	−0.0068 (14)	0.0055 (15)	−0.0012 (14)
C36	0.0274 (18)	0.034 (2)	0.040 (2)	−0.0056 (15)	0.0046 (15)	−0.0016 (16)
C25	0.0341 (19)	0.042 (2)	0.0278 (19)	0.0046 (16)	0.0088 (15)	0.0031 (17)
C35	0.0263 (18)	0.044 (2)	0.045 (2)	−0.0093 (17)	−0.0015 (16)	0.0039 (19)
C7	0.034 (2)	0.052 (2)	0.033 (2)	−0.0089 (18)	0.0145 (16)	−0.0053 (18)
C2	0.0327 (19)	0.0305 (19)	0.034 (2)	0.0044 (15)	−0.0007 (15)	0.0015 (16)
C24	0.036 (2)	0.036 (2)	0.054 (3)	−0.0064 (17)	0.0154 (18)	0.0106 (19)
C16	0.038 (2)	0.034 (2)	0.050 (2)	0.0086 (17)	0.0088 (18)	0.0113 (19)
C3	0.040 (2)	0.047 (2)	0.044 (2)	0.0149 (19)	0.0039 (18)	−0.009 (2)
C15	0.037 (2)	0.056 (3)	0.044 (2)	0.011 (2)	0.0034 (18)	0.024 (2)
C34	0.034 (2)	0.082 (3)	0.050 (3)	−0.011 (2)	0.0230 (19)	−0.001 (2)
C14	0.067 (3)	0.074 (3)	0.030 (2)	0.003 (3)	−0.003 (2)	−0.002 (2)
O1	0.088 (3)	0.072 (2)	0.0390 (17)	−0.0155 (19)	0.0133 (16)	0.0106 (16)
O2	0.141 (15)	0.087 (9)	0.041 (7)	0.065 (10)	0.020 (8)	−0.001 (6)
O3	0.093 (7)	0.060 (8)	0.066 (7)	−0.046 (6)	−0.006 (5)	0.026 (6)
O4	0.023 (5)	0.143 (13)	0.112 (11)	0.011 (6)	0.010 (5)	−0.008 (8)
O4'	0.098 (11)	0.158 (16)	0.066 (8)	−0.059 (10)	−0.012 (7)	0.035 (9)
O3'	0.23 (2)	0.041 (7)	0.106 (12)	0.035 (8)	0.036 (13)	−0.011 (6)
O2'	0.097 (9)	0.111 (14)	0.085 (12)	0.045 (9)	0.065 (9)	0.029 (8)

### Geometric parameters (Å, º)

**Table:**

Cu1—Cl1	2.2698 (10)	N4—C3	1.461 (5)
Cu1—N21	1.976 (3)	C12—C5	1.491 (5)
Cu1—N1	2.137 (3)	C26—H26	0.9300
Cu1—N31	2.041 (3)	C26—C25	1.349 (5)
Cu1—N11	2.173 (3)	C22—C6	1.474 (5)
Cl2—O1	1.428 (3)	C5—H5	0.9800
Cl2—O2	1.395 (10)	C32—C7	1.503 (5)
Cl2—O3	1.402 (8)	C6—H6A	0.9700
Cl2—O4	1.396 (9)	C6—H6B	0.9700
Cl2—O4'	1.410 (11)	C36—H36	0.9300
Cl2—O3'	1.410 (10)	C36—C35	1.341 (5)
Cl2—O2'	1.405 (10)	C25—H25	0.9300
N21—C26	1.378 (4)	C35—H35	0.9300
N21—C22	1.320 (4)	C7—H7A	0.9700
N1—C5	1.501 (4)	C7—H7B	0.9700
N1—C6	1.497 (4)	C2—H2A	0.9700
N1—C2	1.503 (4)	C2—H2B	0.9700
N31—C32	1.331 (4)	C2—C3	1.530 (5)
N31—C36	1.381 (4)	C24—H24A	0.9600
N23—C22	1.343 (4)	C24—H24B	0.9600
N23—C25	1.370 (5)	C24—H24C	0.9600
N23—C24	1.452 (5)	C16—H16	0.9300
N11—C12	1.318 (5)	C16—C15	1.339 (6)
N11—C16	1.368 (5)	C3—H3A	0.9700
N33—C32	1.355 (4)	C3—H3B	0.9700
N33—C35	1.366 (5)	C15—H15	0.9300
N33—C34	1.473 (4)	C34—H34A	0.9600
N13—C12	1.348 (4)	C34—H34B	0.9600
N13—C15	1.377 (5)	C34—H34C	0.9600
N13—C14	1.466 (5)	C14—H14A	0.9600
N4—C5	1.445 (4)	C14—H14B	0.9600
N4—C7	1.455 (5)	C14—H14C	0.9600
			
N21—Cu1—Cl1	91.82 (9)	N1—C5—H5	108.5
N21—Cu1—N1	80.47 (11)	N4—C5—N1	106.5 (3)
N21—Cu1—N31	147.65 (12)	N4—C5—C12	116.3 (3)
N21—Cu1—N11	113.64 (12)	N4—C5—H5	108.5
N1—Cu1—Cl1	171.21 (8)	C12—C5—N1	108.4 (3)
N1—Cu1—N11	77.43 (11)	C12—C5—H5	108.5
N31—Cu1—Cl1	95.04 (9)	N31—C32—N33	110.0 (3)
N31—Cu1—N1	93.74 (11)	N31—C32—C7	129.8 (3)
N31—Cu1—N11	95.74 (11)	N33—C32—C7	120.0 (3)
N11—Cu1—Cl1	102.11 (8)	N1—C6—H6A	110.3
O2—Cl2—O1	108.7 (10)	N1—C6—H6B	110.3
O2—Cl2—O3	111.3 (11)	C22—C6—N1	107.3 (3)
O2—Cl2—O4	110.3 (11)	C22—C6—H6A	110.3
O2—Cl2—O4'	94.5 (15)	C22—C6—H6B	110.3
O2—Cl2—O3'	120.9 (15)	H6A—C6—H6B	108.5
O2—Cl2—O2'	16 (2)	N31—C36—H36	125.3
O3—Cl2—O1	106.8 (7)	C35—C36—N31	109.4 (3)
O3—Cl2—O4'	125.1 (12)	C35—C36—H36	125.3
O3—Cl2—O3'	18.1 (13)	N23—C25—H25	126.4
O3—Cl2—O2'	96.2 (15)	C26—C25—N23	107.1 (3)
O4—Cl2—O1	110.3 (9)	C26—C25—H25	126.4
O4—Cl2—O3	109.3 (10)	N33—C35—H35	126.5
O4—Cl2—O4'	18.1 (16)	C36—C35—N33	107.0 (3)
O4—Cl2—O3'	91.3 (13)	C36—C35—H35	126.5
O4—Cl2—O2'	121.4 (15)	N4—C7—C32	120.9 (3)
O4'—Cl2—O1	109.4 (7)	N4—C7—H7A	107.1
O3'—Cl2—O1	113.8 (9)	N4—C7—H7B	107.1
O3'—Cl2—O4'	107.5 (11)	C32—C7—H7A	107.1
O2'—Cl2—O1	111.1 (12)	C32—C7—H7B	107.1
O2'—Cl2—O4'	107.4 (12)	H7A—C7—H7B	106.8
O2'—Cl2—O3'	107.5 (12)	N1—C2—H2A	111.0
C26—N21—Cu1	138.6 (2)	N1—C2—H2B	111.0
C22—N21—Cu1	114.2 (2)	N1—C2—C3	104.0 (3)
C22—N21—C26	106.5 (3)	H2A—C2—H2B	109.0
C5—N1—Cu1	110.19 (19)	C3—C2—H2A	111.0
C5—N1—C2	105.7 (2)	C3—C2—H2B	111.0
C6—N1—Cu1	107.06 (19)	N23—C24—H24A	109.5
C6—N1—C5	109.8 (2)	N23—C24—H24B	109.5
C6—N1—C2	113.2 (3)	N23—C24—H24C	109.5
C2—N1—Cu1	110.9 (2)	H24A—C24—H24B	109.5
C32—N31—Cu1	134.3 (2)	H24A—C24—H24C	109.5
C32—N31—C36	106.1 (3)	H24B—C24—H24C	109.5
C36—N31—Cu1	119.2 (2)	N11—C16—H16	124.8
C22—N23—C25	107.1 (3)	C15—C16—N11	110.4 (4)
C22—N23—C24	125.8 (3)	C15—C16—H16	124.8
C25—N23—C24	127.1 (3)	N4—C3—C2	106.9 (3)
C12—N11—Cu1	111.1 (2)	N4—C3—H3A	110.3
C12—N11—C16	105.5 (3)	N4—C3—H3B	110.3
C16—N11—Cu1	141.8 (3)	C2—C3—H3A	110.3
C32—N33—C35	107.5 (3)	C2—C3—H3B	110.3
C32—N33—C34	128.3 (3)	H3A—C3—H3B	108.6
C35—N33—C34	124.2 (3)	N13—C15—H15	126.9
C12—N13—C15	106.8 (3)	C16—C15—N13	106.2 (3)
C12—N13—C14	127.2 (3)	C16—C15—H15	126.9
C15—N13—C14	126.0 (3)	N33—C34—H34A	109.5
C5—N4—C7	118.8 (3)	N33—C34—H34B	109.5
C5—N4—C3	103.6 (3)	N33—C34—H34C	109.5
C7—N4—C3	116.4 (3)	H34A—C34—H34B	109.5
N11—C12—N13	111.2 (3)	H34A—C34—H34C	109.5
N11—C12—C5	122.0 (3)	H34B—C34—H34C	109.5
N13—C12—C5	126.9 (3)	N13—C14—H14A	109.5
N21—C26—H26	125.7	N13—C14—H14B	109.5
C25—C26—N21	108.5 (3)	N13—C14—H14C	109.5
C25—C26—H26	125.7	H14A—C14—H14B	109.5
N21—C22—N23	110.7 (3)	H14A—C14—H14C	109.5
N21—C22—C6	121.3 (3)	H14B—C14—H14C	109.5
N23—C22—C6	127.9 (3)		
			
Cu1—N21—C26—C25	−169.6 (3)	C5—N4—C3—C2	33.7 (4)
Cu1—N21—C22—N23	172.2 (2)	C32—N31—C36—C35	−0.9 (4)
Cu1—N21—C22—C6	−3.5 (4)	C32—N33—C35—C36	−0.1 (4)
Cu1—N1—C5—N4	−94.3 (2)	C6—N1—C5—N4	148.0 (3)
Cu1—N1—C5—C12	31.5 (3)	C6—N1—C5—C12	−86.2 (3)
Cu1—N1—C6—C22	30.9 (3)	C6—N1—C2—C3	−124.7 (3)
Cu1—N1—C2—C3	114.9 (3)	C36—N31—C32—N33	0.8 (4)
Cu1—N31—C32—N33	−171.5 (2)	C36—N31—C32—C7	175.2 (4)
Cu1—N31—C32—C7	3.0 (6)	C25—N23—C22—N21	0.4 (4)
Cu1—N31—C36—C35	172.8 (3)	C25—N23—C22—C6	175.8 (3)
Cu1—N11—C12—N13	169.3 (2)	C35—N33—C32—N31	−0.4 (4)
Cu1—N11—C12—C5	−11.4 (4)	C35—N33—C32—C7	−175.5 (3)
Cu1—N11—C16—C15	−163.7 (3)	C7—N4—C5—N1	94.3 (3)
N21—C26—C25—N23	0.4 (4)	C7—N4—C5—C12	−26.5 (4)
N21—C22—C6—N1	−20.1 (4)	C7—N4—C3—C2	−98.7 (4)
N1—C2—C3—N4	−17.7 (4)	C2—N1—C5—N4	25.6 (3)
N31—C32—C7—N4	38.0 (6)	C2—N1—C5—C12	151.4 (3)
N31—C36—C35—N33	0.6 (4)	C2—N1—C6—C22	−91.6 (3)
N23—C22—C6—N1	165.0 (3)	C24—N23—C22—N21	−179.5 (3)
N11—C12—C5—N1	−13.4 (4)	C24—N23—C22—C6	−4.2 (5)
N11—C12—C5—N4	106.4 (4)	C24—N23—C25—C26	179.4 (3)
N11—C16—C15—N13	0.8 (4)	C16—N11—C12—N13	0.5 (4)
N33—C32—C7—N4	−148.0 (3)	C16—N11—C12—C5	179.8 (3)
N13—C12—C5—N1	165.8 (3)	C3—N4—C5—N1	−36.7 (3)
N13—C12—C5—N4	−74.4 (4)	C3—N4—C5—C12	−157.5 (3)
C12—N11—C16—C15	−0.8 (4)	C3—N4—C7—C32	54.2 (5)
C12—N13—C15—C16	−0.5 (4)	C15—N13—C12—N11	0.0 (4)
C26—N21—C22—N23	−0.1 (4)	C15—N13—C12—C5	−179.3 (3)
C26—N21—C22—C6	−175.9 (3)	C34—N33—C32—N31	−178.2 (4)
C22—N21—C26—C25	−0.2 (4)	C34—N33—C32—C7	6.7 (6)
C22—N23—C25—C26	−0.5 (4)	C34—N33—C35—C36	177.8 (4)
C5—N1—C6—C22	150.5 (3)	C14—N13—C12—N11	178.4 (4)
C5—N1—C2—C3	−4.5 (4)	C14—N13—C12—C5	−0.9 (6)
C5—N4—C7—C32	−70.9 (5)	C14—N13—C15—C16	−178.9 (4)
